# Alkaline Phosphatases Account for Low Plasma Levels of Inorganic Pyrophosphate in Chronic Kidney Disease

**DOI:** 10.3389/fcell.2020.586831

**Published:** 2020-12-03

**Authors:** Audrey Laurain, Isabelle Rubera, Christophe Duranton, Frank Rutsch, Yvonne Nitschke, Elodie Ray, Sandor Vido, Antoine Sicard, Georges Lefthériotis, Guillaume Favre

**Affiliations:** ^1^Faculty of Medicine, Côte d’Azur University, Nice, France; ^2^UMR 7073, Laboratory of Physiology and Molecular Medicine (LP2M), Centre National de la Recherche Scientifique, Nice, France; ^3^Nephrology Department, University Hospital, Nice, France; ^4^Department of General Pediatrics, Muenster University Children’s Hospital, Muenster, Germany; ^5^Department of Vascular Medicine and Surgery, University Hospital, Nice, France

**Keywords:** alkaline phosphatase activity, hemodialysis, kidney transplant, pyrophosphate, purinergic mechanisms, mineral and bone disorder (CKD-MBD)

## Abstract

**Introduction:**

Patients on dialysis and kidney transplant recipients (KTR) present the syndrome of mineral and bone disorders (MBD), which share common traits with monogenic calcifying diseases related to disturbances of the purinergic system. Low plasma levels of inorganic pyrophosphate (PP_i_) and ectopic vascular calcifications belong to these two conditions. This suggests that the purinergic system may be altered in chronic kidney disease with MBD. Therefore, we perform a transversal pilot study in order to compare the determinants of PPi homeostasis and the plasma levels of PPi in patients on dialysis, in KTR and in healthy people.

**Patients and Methods:**

We included 10 controls, 10 patients on maintenance dialysis, 10 early KTR 3 ± 1 months after transplantation and nine late KTR 24 ± 3 months after transplantation. We measured aortic calcifications, plasma and urine levels of PP_i_, the renal fractional excretion of PP_i_ (FePP_i_), nucleoside triphosphate hydrolase (NPP) and ALP activities in plasma. Correlations and comparisons were assessed with non-parametric tests.

**Results:**

Low PP_i_ was found in patients on dialysis [1.11 (0.88–1.35), *p* = 0.004], in early KTR [0.91 (0.66–0.98), *p* = 0.0003] and in late KTR [1.16 (1.07–1.45), *p* = 0.02] compared to controls [1.66 (1.31–1.72) μmol/L]. Arterial calcifications were higher in patients on dialysis than in controls [9 (1–75) vs. 399 (25–526) calcium score/cm^2^, *p* < 0.05]. ALP activity was augmented in patients on dialysis [113 (74–160), *p* = 0.01] and in early KTR [120 (84–142), *p* = 0.002] compared to controls [64 (56–70) UI/L]. The activity of NPP and FePP_i_ were not different between groups. ALP activity was negatively correlated with PP_i_ (*r* = −0.49, *p* = 0.001).

**Discussion:**

Patients on dialysis and KTR have low plasma levels of PP_i_, which are partly related to high ALP activity, but neither to low NPP activity, nor to increased renal excretion of PP_i_. Further work is necessary to explore comprehensively the purinergic system in chronic kidney disease.

## Introduction

Patients with chronic kidney disease (CKD) and those receiving renal replacement therapy exhibit various traits of the syndrome of mineral and bone disorders (MBD) during their lifetime (Ketteler et al., 2017). This syndrome is characterized by ectopic calcifications located in the intimal or medial arterial layers (Vervloet and Cozzolino, 2017) increasing the risk of cardiovascular-related events and mortality (Chiu et al., 2010). It is also characterized by low plasma levels of calcium, high plasma levels of inorganic phosphates (P_i_) and of parathyroid hormone (PTH), and by an increased alkaline phosphatase (ALP) plasma activity (Stevens et al., 2006). Bone turn-over ranges from high to low (Kurz et al., 1994) in relation to decreased bone mineral density observed in approximately 50% of patients, resulting in a higher risk of bone fracture (Miller et al., 2005). Any combination of the aforementioned abnormalities depends on the course and management of the renal disease (Miller et al., 2005; Stevens et al., 2006; Mazzaferro et al., 2009; Chiu et al., 2010; Wolf et al., 2016).

Here, we consider inorganic pyrophosphate (PP_i_) in patients with end stage CKD on renal replacement therapies as a new player in the syndrome of MBD. Indeed, PP_i_ is one of the main circulating endogenous calcification inhibitors (Back et al., 2018). Micromoles of PP_i_ appear sufficient to inhibit the deposition of hydroxyapatite crystal on collagen activating sites in the presence of millimoles of Ca and P_i_ (Fleisch et al., 1966). In patients on maintenance dialysis [PP_i_]pl is low for unknown reasons (Lomashvili et al., 2005; O’Neill et al., 2010). Since PP_i_ is hydrolyzed by ALP, this might be due to high ALP, because a 30% decrease of [PP_i_]pl is observed at the end of the dialysis session in conjunction with significantly increased plasma ALP activity (Azpiazu et al., 2018) and because high plasma ALP activity belongs to the syndrome of MBD in end stage CKD (Kurz et al., 1994; Wallace et al., 1996; Wolf et al., 2016; Haarhaus et al., 2017; Ketteler et al., 2017; Bover et al., 2018). Inorganic pyrophosphate is a key compound in the purinergic system. Actually, PP_i_ homeostasis has been shown to primarily depend on adenosine triphosphate (ATP) metabolism. Levels of extracellular PP_i_ are partly regulated by the ATP-binding cassette transporter, subfamily C, member 6 (ABCC6) (Le Saux et al., 2000) and by ectonucleotide pyrophosphatase/phosphodiesterase 1 (ENPP1) (Rutsch et al., 2003). The liver is the main source of ABCC6-mediated and ENPP1-mediated extracellular PPi (Jansen et al., 2014). Another source of PP_i_ is the secretion from its intracellular pool into the extracellular fluid by inorganic pyrophosphate transport regulator (ANKH) (Netter et al., 2004; Mitton-Fitzgerald et al., 2016). The influence of the diet on plasma PPi levels is very limited (Dedinszki et al., 2017; Pomozi et al., 2017). Finally, circulating PP_i_ is hydrolyzed by ALP and eliminated in urine (Letavernier et al., 2019).

A disruption in the balance between PP_i_ production and degradation results in disturbed mineralization process and low [PP_i_]pl in human with disorders of the purinergic system (Fish et al., 2013) and very likely in CKD. Therefore, we explored the relationship between [PP_i_]_pl_ and the main compounds of the purinergic system in patients with CKD and MBD.

## Patients and Methods

### Patient Recruitment

**FIGURE 1:**
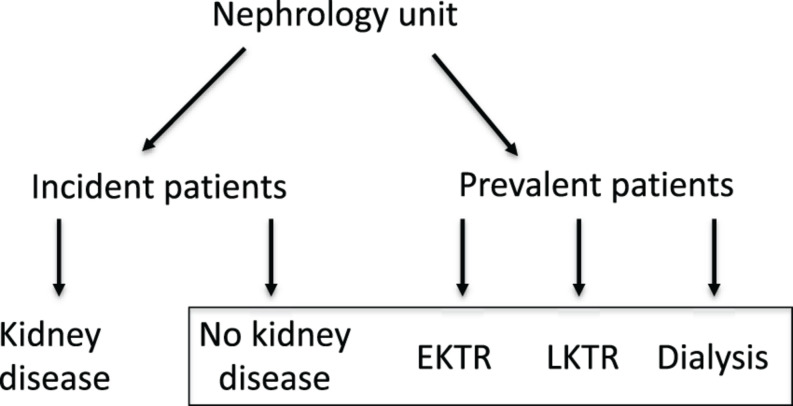
Workflow (selection of healthy people and patients).

For this cross-sectional pilot study, we included patients with well-defined phenotypes and controls (Figure 1). We selected 10 controls with no renal impairment, i.e., eGFR > 60 ml/min/1.73 m^2^, no albuminuria, normal red and white urinary cell counts and normal renal ultrasonography. We selected patients on maintenance therapy for end-stage chronic kidney disease: 10 patients on maintenance hemodialysis (HD) and on a waiting list for KT; 10 KT recipients at 2 ± 1 months post-transplantation or early KTR (EKTR); nine KT recipients at 24 ± 3 months post-transplantation or late KTR (LKTR). All patients and controls gave informed consent for the study and underwent routine medical examination and fasting blood and urine analysis. Computer tomogram (CT) scans were collected when available but were not performed for the purpose of the study. The study was approved by the institutional Ethics Committee.

### Biochemical and Hormonal Assays

#### PPi

Pyrophosphate was measured in plasma and urine using an enzyme assay. Patient blood samples were collected in the morning after an overnight fasting period (venous punction) directly in a tube containing citrate, theophyllin, adenosine and dypiridamole (CTAD). Plasmas were ultra-filtrated at 4°C using centrisart filtration units (300 KDa cut-off, Sartorius) according to Tolouian et al. (2012). Urine samples were collected in the morning concomitantly to the blood samples. PPi quantifications were performed using a modified method based on the enzyme assay described by Jansen et al. (2014).

Briefly, this method is based on the conversion of PP_i_ into ATP with ATP sulfurylase (Perkin Elmer, Boston MA, United States) and ATP concentrations are measured using a luminescent ATP detection kit (ATPlite^®^, PerkinElmer). The values were corrected using the basal ATP levels measured in each sample. Dosage accuracy was 10% with 0.12 μmol/L absolute uncertainty.

#### Nucleoside Triphosphate Hydrolase (NPP) Activity

Enzyme activity was measured in heparinized plasma, buffered 1:2 in 0.2 M Tris, 1.6 mM MgCl_2_, pH 8.1 by a previously described colorimetric assay using the synthetic substrate p-nitrophenylthymidine 5’-monophosphate (PNTM) (Rutsch et al., 2003). Protein concentration was determined using Pierce^TM^ BCA^TM^ Protein-Assay (Thermo Fisher Scientific). NPP activity was defined as 1 μmol substrate hydrolyzed per μg protein per hour.

Serum creatinine was assessed with Jaffe’s kinetic methods (IDMS standardized), serum calcium was measured by absorption spectrophotometry, serum Pi was measured with UV methods on Cobas 8000 (RocheDiagnostics, Mannheim, Germany). 25-hydroxy vitamin D (25-OH vit D) was assessed by immunologic luminescence (Diasorin^®^, Fallugia, Italy). Plasma ALP activity was assessed by absorbance at 450 nm of paranitrophenol at alkaline pH. Bone ALP isoform was measured using an ^125^I-labeled sandwich radioimmunoassay (Immunotech, Beckman-Coulter Society, Marseille, France). Bio-intact parathyroid hormone (PTH) was measured by luminescence immunoassay (Advia Centaur Intact PTH, Siemens healthcare, Tarrytown, United States). Osteocalcin was assessed by immunoassay (ECLIA, RocheDiagnostics, Mannheim, Germany) and luminescence was determined with an automated device (Liaison XL, Diasorin^®^, Fallugia, Italy).

### Measurement of Arterial Calcification

Arterial calcification was measured on CT scans. Data were reconstructed with Aquarius^©^ software (Tera Recon, San Mateo, CA, United States) to obtain 3-mm-thick consecutive slices. Calcification was measured with the open source Horos Software 2.0 between the right renal artery and the distal abdominal aorta according to the method described by Agatston et al. (1990). Arterial surface was computed from the length (L) and the mean value of three diameters (D) according to the following formula: L × D ×π (in cm^2^). Results were expressed as calcium score divided by the surface of this segment of the abdominal aorta (calcium score/cm^2^).

#### Calculations

GFR was estimated (eGFR) using the CKD-EPI formula according to the plasma creatinine level. Body mass index was calculated as weight in kg divided by height in meters squared (kg/m^2^). Fractional excretion of PP_i_ (FePP_i_) was calculated as a percentage according to the following formula: [PP_i_]_u_ × [creat]_pl__/_[PPi]_pl_ × [creat]_u_ × 100.

#### Statistics

Data are presented as medians with interquartile ranges [25th–75th percentiles]. All statistics were conducted with GraphPad Prism 6^®^ software. Data were compared using non-parametric tests: Kruskal Wallis test, followed by a Mann Whitney test in case of a statistically significant difference. Correlations were evaluated with Spearman’s test. A *p* < 0.05 was considered statistically significant.

## Results

**FIGURE 2:**
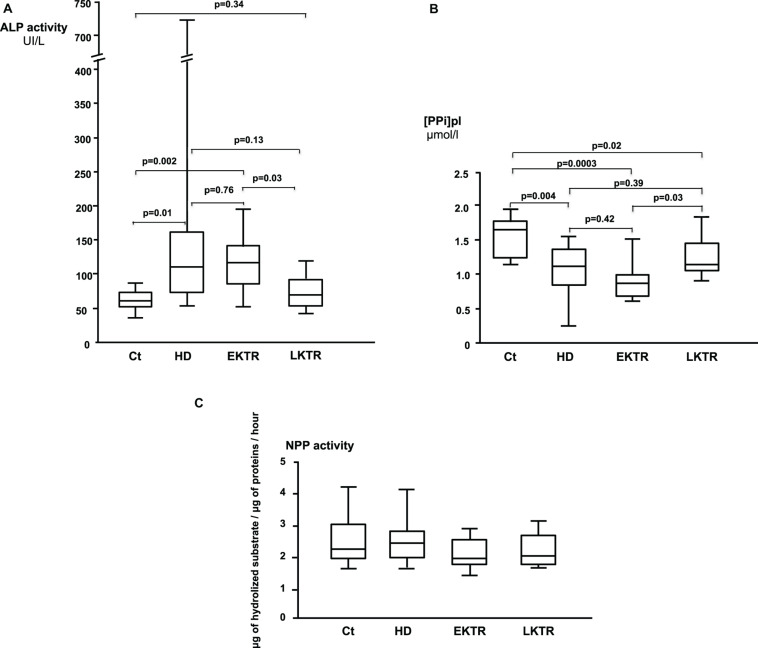
**(A)** Plasma ALP activity (Median, IQR, and highest and lowest values). **(B)** Plasma levels of PP_i_ (Median, IQR, and highest and lowest values). **(C)** Plasma activities of NPP (Median, IQR, and highest and lowest values). There were no statistically significant differences.

**FIGURE 3:**
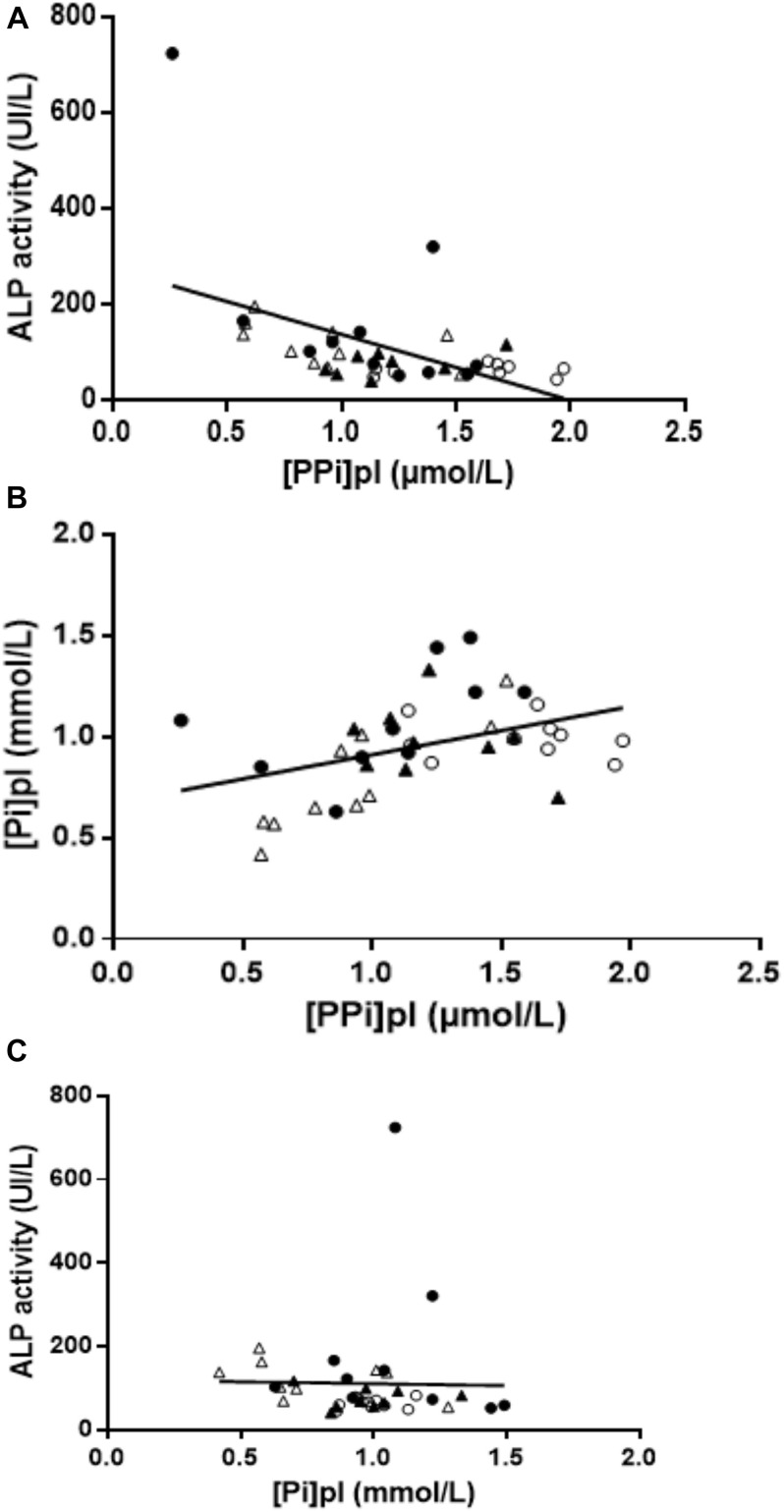
**(A)** Relationship between plasma ALP activity and [PPi]pl. Negative correlation between plasma ALP activity and [PPi]pl (*r* = −0.49, *p* = 0.001). Empty circles = controls; dark circles = HD; empty triangle = EKTR; dark triangle = LKTR. **(B)** Relationship between PP_i_ and P_i_. Positive correlation between [PP_i_]pl and [P_i_]pl (*r* = 0.43, *p* = 0.006). Empty circles = controls; dark circles = HD; empty triangle = EKTR; dark triangle = LKTR. **(C)** Relationship between plasma ALP activity and [Pi]pl. There was no correlation between [P_i_]pl and ALP activity (*r* = −0.23, *p* = 0.16). Empty circles = controls; dark circles = HD; empty triangle = EKTR; dark triangle = LKTR.

Overall, 39 patients were included in our study (Table 1). Age range and BMI were comparable in all groups. Hypertension was present in 40% of controls, 60% of HD, 40% of EKTR and 22% of LKTR. Type 2 diabetes (DT2) was present in 10% of controls, 30% of HD, 30% of EKTR whereas LKTR did not have DT2. Dyslipidemia was present in 30% of controls, 70% of HD, 60% of EKTR and 44% of LKTR. The syndrome of MBD was reflected by the degree of arterial calcification, which was significantly higher in HD than in controls; by significantly higher levels of parathyroid hormone (PTH) and osteocalcin in HD and KTR than in controls; by significantly higher bone ALP isoforms (Table 1) and higher ALP activity (Figure 2A) in HD or EKTR than in controls. Of note, two patients had very high ALP activity due to their poor compliance with treatment. All HD but one received phosphate binders and eight took 1-alpha 25-OH Vit D while most patients received cholecalciferol. Plasma levels of PP_i_ were significantly lower in all patient groups than in controls (Figure 2B). In contrast, renal elimination of PP_i_ was similar in all groups according to the FePP_i_ (Table 1). Plasma NPP activity was the same in all groups (Figure 2C). Plasma ALP activity was strongly and negatively correlated to [PP_i_]pl (Figure 3A) and [PP_i_]pl was positively correlated to [P_i_]pl (Figure 3B). In contrast, there was no significant correlation between plasma ALP activity and [P_i_]pl (Figure 3C) nor between plasma ALP activity and CRP levels (*r* = 0.28, *p* = 0.09), nor between bone ALP isoforms and CRP levels (*r* = 0.18; *p* = 0.27).

**TABLE 1:** Patient and mineral and bone disorder characteristics.

	**Ct (*n* = 10)**	**HD (*n* = 10)**	**EKTR (*n* = 10)**	**LKTR (*n* = 9)**
Age (years)	58 [47–68]	58 [54–64]	59 [44–72]	50 [37–59]
Sex ratio (M/F)	7/3	4/6	5/5	4/6
Hypertension	4 (40%)	6 (60%)	4 (40%)	2 (22%)
Type 2 diabetes	1 (10%)	4 (40%)	3 (30%)	0 (%)
Dyslipidemia	2 (20%)	7 (70%)	6 (60%)	4 (44%)
BMI (kg/m^2^)	28 [26–32]	27 [22–32]	26 [24–29]	25 [22–28]
eGFR (mL/min/1.73 m^2^)	70 [64–74]	–	44 [38–47]**	58 [50–68]
PTH (pg/mL)	55 [46–62]	300 [234–504]**	188 [153–210]**	134 [89–143]**
25-OH Vit D (ng/mL)	38 [30–45]	45 [33–50]	32 [26–42]	43 [28–48]
Bone specific alkaline phosphatase (μg/L)	9.2 [5.8–12.7]	15.5 [10.5–28.1]*	25.9 [14.9–31.1]**	5.7 [5.2–6.3]
[Ca]pl (mmol/L)	2.41[2.33–2.42]	2.20 [2.14–2.24]**	2.40 [2.34–2.51]	2.49 [2.32–2.52]
[P_i_]pl (mmol/L)	0.99 [0.95–1.03]	1.06 [0.91–1.22]	0.69 [0.60–0.99]	0.97 [0.86–1.04]
Osteocalcin (μg/L)	22.6 [20.9–26.8]	137.0 [100.7–263.8]**	46.5 [25.2–83.0]**	32.5 [26.3–47.9]*
Arterial calcifications (calcium score/cm^2^)	9 [1–75]	399 [250–526]*	nd	nd
Fe PP_i_ (%)	11 [4–13%]	nd	14 [9–31%]	9 [8–16%]

*Vs ctrl P < 0.05. **P < 0.01.

All incident or prevalent patients were asked to take part to the study unless they had an acute co-existing pathology, were below 18 years old or were incapable of giving informed consent. The inclusion of 10 patients in each group was anticipated and the patients could withdraw their consent anytime.

Patients without kidney disease after comprehensive examination were called controls. Early kidney transplant recipients (EKTR) were included at 2 ± 1 months post-transplantation. Late kidney transplant recipients (LKTR) were included at 24 ± 3 months post-transplantation. Routine medical examination and fasting blood and urine analysis were performed on a dedicated consultation for the purpose of the study.

## Discussion

In the present study, we observed that [PP_i_]pl levels were negatively correlated to plasma ALP activity and that [PP_i_]pl was low in HD and remained low in KTR despite normalization of ALP activity, indicating that several mechanisms were controlling PP_i_ homeostasis in addition to ALP.

High plasma ALP activity was a major determinant for low [PP_i_]pl in our study (Figure 2A). This was expected for the following reasons: 1/the elimination of PP_i_ depends on its hydrolysis by tissue non-specific alkaline phosphatase (TNAP), encoded by the *ALPL* gene (Favre et al., 2017; Whyte, 2017), 2/ALP activity increases at the end of a dialysis session as compared to its baseline level and this results in 30% decrease of [PPi]pl (Azpiazu et al., 2018), 3/pharmacological blockade of ALP activity restored [PP_i_]pl in uremic mice (Tani et al., 2020). Plasma ALP activity and plasma levels of bone ALP isoforms followed the same pattern in our patients, in accordance with the overproduction of TNAP in CKD (Bover et al., 2018; Nizet et al., 2020). In contrast to our findings, Lomashvili et al. observed no correlation between plasma ALP activity and [PP_i_]pl among 38 patients on maintenance dialysis (Lomashvili et al., 2005). This discrepancy might result from the broader range of plasma ALP activity in our cohort, including values both within and above normal limits. Since we found no statistically significant relationship between ALP activity and CRP, in contrast to published reports (Haarhaus et al., 2017), the degree of inflammation did not appear to influence ALP activity nor bone ALP isoforms in our patients. Here, high ALP activity belonged to the syndrome of MBD because high plasma ALP activity matched elevated bone ALP isoforms, was associated with high levels of osteocalcin and PTH (Table 1), as already described (Kurz et al., 1994; Wolf et al., 2016), and with the presence of arterial calcifications, in line with the literature (London et al., 2005).

We observed a positive correlation between [PP_i_]pl and [P_i_]pl (Figure 2C), which is consistent with other reports from three independent cohorts of patients on maintenance dialysis (Lomashvili et al., 2005; O’Neill et al., 2010; Villa-Bellosta et al., 2016). This led to the hypothesis that [P_i_]pl could exert a negative feedback on ALP activity according to data observed *ex vivo* (Fernley and Walker, 1967; Coburn et al., 1998). However, our data were not in favor of such a negative feedback of [P_i_]pl on ALP activity, as the latter was not correlated to [P_i_]pl (Figure 2B). Consequently, we suggest that this balance may depend on undetermined cellular processes.

We observed that KT did not result in normal levels of [PP_i_]pl 2 years after renal graft, despite recovery of a normal glomerular filtration rate and restoration of ALP activity to normal levels. Indeed, shortly after KT (2 ± 1 months), the low [PP_i_]pl was in line with enhanced hydrolysis of PP_i_ due to raised plasma ALP activity. However, 2 years after KT, plasma ALP activity was normal and could not explain low [PP_i_]pl levels. In light of the knowledge derived from studies of generalized arterial calcification of infancy (GACI), a rare disease characterized by low [PP_i_]pl due to an inactivating mutation of *ENPP1* (Rutsch et al., 2003), we measured NPP plasma activity which was within normal values (Figure 1). Since PP_i_ is excreted in urine, we measured its renal elimination as reflected by FePP_i_ and found no increased PP_i_ excretion (Table 1). According to the mechanisms involved in pseudoxanthoma elasticum, due to mutations in *ABCC6* accounting for low [PP_i_]pl (Le Saux et al., 2000) and vascular calcification (Leftheriotis et al., 2013), it could be that the production of PP_i_ related to *ABCC6* was decreased in our patients. Indeed, experimental data from uremic rats showed that low [PP_i_]pl resulted from low *ABCC6* protein expression in the kidneys and in the liver (Lau et al., 2014). Furthermore, ANKH controls [PP_i_]pl levels in uremic rats (Zhao et al., 2012; Chen et al., 2019). However, in humans, mutations of *ANKH* account for craniometaphyseal dysplasia and, to the best of our knowledge, [PP_i_]pl levels was not yet measured in this condition (Chen et al., 2011).

This study is the first one, to the best of our knowledge, exploring the crosstalk between the purinergic system and mineral and bone disorder in CKD. The low [PPi]pl in KTR and the inverse relationship between ALP activity and [PPi]pl are new observations. However, this study is limited by the low number of patients and by the lack of association between biological and clinical data. Larger studies are needed to confirm our observations.

## Conclusion

In conclusion, low [PP_i_]pl is partly related to its high level of hydrolysis due to elevated plasma ALP activity in patients with MBD induced by CKD. This is not the only mechanism because [PP_i_]pl remains low although plasma ALP activity is restored to normal levels after kidney transplantation. In addition to many experimental data, our pilot clinical study suggests that the purinergic system should be comprehensively explored in CKD in order to shed light on the complex pathogenesis of MBD (Back et al., 2018).

## Data Availability Statement

The raw data supporting the conclusions of this article will be made available by the authors, without undue reservation, to any qualified researcher.

## Ethics Statement

The studies involving human participants were reviewed and approved by Comite de Protection des Personnes Sud Mediterranee III CHU de Nimes. The patients/participants provided their written informed consent to participate in this study. Written informed consent was obtained from the individual(s) for the publication of any potentially identifiable images or data included in this article.

## Author Contributions

AL selected the patients, performed the clinical and biological investigations, determined the plasma levels of PP_i_. IR and CD set up the dosage’s method for PP_i_, performed the dosages and provided their scientific expertises to the study. FR and YN performed the assays for NPP1 activity and provided their scientific expertises to the study. ER measured the arterial calcifications. SV referred the patients on maintenance dialysis and provided his medical expertise to the study. AS referred the transplanted patients and provided his medical expertise to the study. GL provided the scientific background for the design of the study and validated the measurements of arterial calcifications. GF delineated the protocol of the study and performed the clinical and biological investigations. All authors significantly contribute to the redaction of the manuscript.

## Conflict of Interest

The authors declare that the research was conducted in the absence of any commercial or financial relationships that could be construed as a potential conflict of interest. The handling editor declared a past co-authorship with several of the authors FR, YN, and GL.
